# Shiga toxin producing *Escherichia coli: *identification of non-O157:H7-Super-Shedding cows and related risk factors

**DOI:** 10.1186/1757-4749-2-7

**Published:** 2010-07-09

**Authors:** Andrea Menrath, Lothar H Wieler, Katrin Heidemanns, Torsten Semmler, Angelika Fruth, Nicole Kemper

**Affiliations:** 1 Institute of Animal Breeding and Husbandry, Christian-Albrechts-University, Hermann-Rodewald-Str. 6, 24118 Kiel, Germany; 2 Institute of Microbiology and Epizootics, Veterinary Faculty, Free University, Philippstr. 13, 10115 Berlin, Germany; 3 Robert Koch Institute, National Reference Centre of Salmonella and Other Enteric Pathogens, Burgstraße 37, 38855 Wernigerode, Germany

## Abstract

**Background:**

Shiga toxin producing *Escherichia coli *(STEC) are an important cause of human gastro-enteritis and extraintestinal sequelae, with ruminants, especially cattle, as the major source of infection and reservoir. In this study, the fecal STEC shedding of 133 dairy cows was analyzed over a period of twelve months by monthly sampling with the aim to investigate shedding patterns and risk factors.

**Results:**

Overall, 24.7% (in total 407) of 1,646 fecal samples were tested positive for *stx *by PCR with inner-herd prevalences on the different farms of 11.1% to 32.3%. At individual levels, cows were *stx*-positive on zero to eight consecutive samplings. According to a strictly longitudinal definition of Super-Shedding, in the present study 14 cows were identified as Super-Shedders of non-O157 serotypes.

Significant risk factors for the shedding of STEC were the month of sampling, the number of lactations and days in lactation, the nutritional condition, the somatic cell count and the content of protein in milk. Most notably, the presence of STEC Super-Shedding cows in the herd was a significant risk factor, revealing that STEC Super-Shedding is not restricted to STEC O157:H7 alone.

**Conclusions:**

These data have implications for possible interventions, as removing single non-O157:H7 STEC Super-Shedding cattle from farms would significantly reduce STEC burden.

## Background

Shiga toxin producing *Escherichia coli *(STEC) are an emerging issue for veterinary public health. Their zoonotic relevance is related to the severity of caused diseases and the low dose of ten to 100 bacteria [1]. The highly cytotoxic Shiga toxin combined with other virulence factors causes gastroenteritis and severe sequelae in children with a low, but steady incidence. Most research to date has concentrated on the STEC-serovar O157:H7, but in Germany and other European countries human infections by non-O157:H7 serovars have also been frequently assessed [2-5]. The only consistent difference of these STEC-serovars to apathogenic *Escherichia coli *(*E. coli*) is the possession of Shiga toxin (*stx*) genes. Potentially pathogenic STEC for humans, causing hemorrhagic colitis and mostly possessing the genes for *stx*_2_, *EHEC-hly*_*A*_and *eae*, encoding intimin, a factor of adherence, are so-called enterohemorrhagic *E. coli *(EHEC), but also other STEC can induce serious disease in humans [2,3,6]. Ruminants, and especially cattle, are considered as a natural reservoir of STEC. Extended risks originate in domesticated cattle kept in close contact to humans. Furthermore, cattle-derived foods can be contaminated and represent an important source of infection as well [7,8]. Bovine carriers of STEC show no sign of clinical disease whilst shedding this pathogen.

Risk factors for fecal shedding by dairy cattle are varied, including different factors of management such as hygiene, the kind of diet, husbandry and changes in housing or structure of the herd [9-11]. Moreover, at least for the serovar O157:H7, the individual characteristics of each cow also seem to have an influence [12]. Based on data from Synge et al., Matthews et al. formed a model simulating the excretion- and transmission-dynamics of STEC O157:H7 in a cattle herd [13-15]. The model fit best to the real data if a small proportion of cows had a 50-fold higher excretion of STEC O157:H7 compared to the other animals [14,15]. Up to 80.0% of the transmissions of STEC O157:H7 to uninfected cows originated in 20.0% of the most infectious cows [14]. These high-level STEC shedding cows were defined as Super-Shedders [12,15]. A high-level excretion of STEC as shown by these Super-Shedders is associated with the colonization of the recto-anal junction of their gastrointestinal tract [12,15,16].

According to the definition of Chase-Topping et al., all cows that shed at least 10^4 ^cfu (colony forming units) STEC O157:H7 per gram feces were characterized as Super-Shedders [12]. In this definition, the longitudinal component of Super-Shedding was totally neglected. However, the concentration of STEC O157:H7 was shown to be associated with the duration of shedding [17]. Hence, in the present study, as in the investigations of Lim et al. and Carlson et al., the longitudinal part of the definition was used [18,19]. In both studies, cows were defined as Super-Shedders, or alternatively as Persistent-Shedders, if they shed STEC O157:H7 for more than three consecutive months. In this study, the definition of a Super-Shedder was extended as follows: cows were classified as Super-Shedders if at least half of their samples and equal or more than four consecutive samples were *stx-*positive.

The aim of this study was to investigate risk factors in milk production for STEC shedding in general by dairy cattle. Furthermore, the occurrence of possible Super-Shedding cows, shedding not only STEC O157:H7 but also other STEC-serovars, was examined by longitudinal and qualitative sampling over the period of twelve months.

## Results

In 24.7% (407) of all samples, *stx *genes were detected by the screening PCR. Within the herds, the mean *stx *prevalence varied between 11.1% and 32.3%.

Of all cows sampled for more than six months (n = 140), only 12.9% (18 cows) were detected as constantly *stx*-negative. Most of the animals excreted *stx *at least once (45.5%, 55 cows), and 17.1% (24 cows) were classed as *stx-*positive in more than 50% of their samples. In 40.7% (57 cows), the maximal duration of *stx *shedding was one sampling, whereas 29 of these cows were identified as intermittent shedders. They were detected in one to three additional but not consecutive samplings. For 32.9% (46 cows) that shed *stx *in two to three consecutive samplings, these samplings in most of the cases (35 cows) were followed by other short periods of *stx *shedding. Seventeen cows (12.1%) were determined to be *stx*-positive in the screening PCR in four or five consecutive samplings. In total, twelve of these cows had additional periods of shedding intermittently or for short periods. Two cows (1.6%) were characterized by a great number of consecutive *stx*-positive samplings: one cow was determined to be *stx*-positive in seven consecutive samplings, the other even over eight consecutive samplings (Table 1). In total, 14 cows (10.0%) were identified as Super-Shedders according to the definition with positive *stx*-detection in the screening PCR on at least four consecutive samplings and in equal to or more than half of their samples. In the sampling period, STEC-colonies were isolated resulting in 1,105 STEC-isolates from 144 of the 407 *stx*-positive fecal samples. Dominating combinations of virulence genes were *stx*_2 _and *EHEC-hly*_*A*_(434 isolates, 39.3%), and *stx*_1_, *stx*_2 _and *EHEC-hly*_*A*_(311 isolates, 28.1%) (Table 2).

**Table 1:** Maximal number of consecutive *stx*-detection in the feces of all cows, investigated for more than six months (n = 140)

consecutive *stx*-positive samples (monthly sampling)	no. of cows	% of cows
0	18	12.9%
1	57	40.7%
2-3	46	32.9%
4-5	17	12.1%
7-8	2	1.6%

%: percentage

**Table 2:** Prevalences of virulence factors from isolated STEC (n = 1,105 isolates)

*stx*_1_	*stx*_2_	*eae*	*EHEC-hly*_*A*_	no. (%) of pos. Isolates (total: 1,105)	
-	+	-	+	434	(39.3%)
+	+	-	+	311	(28.1%)
-	+	-	-	145	(13.1%)
+	+	-	-	56	(5.1%)
-	+	+	+	43	(3.9%)
+	-	-	-	35	(3.2%)
+	-	-	+	33	(3.0%)
+	-	+	+	24	(2.2%)
+	+	+	+	17	(1.5%)
-	+	+	-	4	(0.4%)
+	+	+	-	2	(0.2%)
+	-	+	-	1	(0.1%)

pos.: positive

%: percentage

From 14 Super-Shedding animals, 380 isolates were identified. From these isolates, 61 STEC-isolates of six Super-Shedders were selected for serotyping based on their virulence pattern, the date of sampling and the cow of origin. To represent each of the four farms with Super-Shedding cows, STEC-isolates from one Super Shedder per farm were chosen. Assuming that on one farm different serovars might be present, STEC-isolates from two additional cows on one of the farms were serotyped. The characterized STEC-isolates belonged to 24 sorbitol-fermenting non-O157:H7 STEC serovars (Table 3). The two most predominant serovars were serovar O113:NM (16 isolates, 26.2%) and O22:H8 (10 isolates, 16.4%), the most frequent virulence patterns of these STEC were *stx*_2 _and *EHEC-hly*_*A *_(39 isolates, 63.9%).

**Table 3:** Serotypes and virulence patterns of tested STEC-isolates (n = 61) from six selected Super-Shedders

		virulence patterns			
			
serovars	no. of isolates	*stx*_2_+, *EHEC*-_*hlyA*_+	*stx*_1_+, stx_2_+, *EHEC*-*hly*_*A*_+	other	**until now isolated according to MicroBioNet **[43]
O113:NM	16	13	2	1 (*stx_2_+*)	cattle, human (D)
O22:H8	10	4	5	1 (*stx*_1_+, *EHEC*-_*hlyA *_+)	cattle, human (D, HUS)
Ont:H25	7	5	1	1 (*stx*_2_+, *eae*+)	cattle, human (D)
O130:H11	6	2	4		cattle
O8:H19	2	2			cattle, human (D, HUS)
O18:H8	2	1		1 (*stx*_1_+, *EHEC*-_*hlyA *_+)	-
O113:H21	2	2			cattle, human (D, HUS, TTP)
O138:H34	2	1		1 (*stx*_2_+)	cattle
O5:H7	1		1		cattle
O8:H21	1			1 (*stx*_1_+)	human (HUS)
O28:H31	1	1			-
O80:H45	1	1			-
O91:H7	1	1			cattle
O150:H8	1		1		cattle
Ont:H7	1			1 (*stx*_2_+)	cattle, human (D)
Ont:H19	1	1			cattle, human (D)
Ont:H21	1	1			cattle, human (D)
Ont:H39	1	1			-
Ont:H42	1	1			cattle
Orough:NM	1	1			cattle, human (D, HUS)
Orough:H2	1			1 (*stx*_1_+, *eae*+, *EHEC*-_*hlyA *_+)**^1^**	cattle, human
Orough:Hnt	1	1			cattle, human (D)

cattle: healthy cattle; human: healthy human; human (D): human case with diarrhea; human (TTP): human case with thrombotic-thrombocytopenic purpura; human (HUS): human case with hemolytic-uremic syndrome

**^1^**: potential EHEC: STEC with virulence pattern *stx_2_+, EHEC-hly_A_+ *and *eae+*

-: no isolation recorded in MicroBioNet [43]

In one cow, the serovar O22:H8 was isolated on each sampling date from July to November. In September, this cow began to shed additionally the serovar O113:NM for the next five consecutive samplings until January. On the same farm, another cow shed the serovar O113:NM over nearly the same period from November to December.

Several risk factors were identified in this study as significant influences on the detection of *stx *in bovine feces: days in milk (DIM) and the number of lactations, the somatic cell count, the content of protein and urea in milk and the nutritional condition. Furthermore, the presence of Super-Shedding cows in the herd and the month of sampling represented significant risk factors. Depending on the DIM class, dry cows, and cows with 50 to 150 days as well as cows with more than 350 days in milk showed a significantly higher risk or a higher tendency to shed STEC (Table 4). First-calving cows, cows with a somatic cell count lower than 100,000 cells/ml milk, a protein content in milk higher than 3.0%, respectively a body condition score higher than 3.50 had a significantly or tendentially increased risk to be identified as STEC-shedders by the screening PCR, whereas cows with an urea content lower than 150 mg/L milk had a decreased risk (Table 4).

**Table 4:** Significant risk factors for shedding of STEC by dairy cattle

variables class	no. of sampled cows	no. of *stx*-pos. cows	% of *stx-*pos. cows	Odds- Ratio	95%-CI	against (Odds = 1)	**sign**.
days in milk							
51.-100.d	175	44	25.1%	1.73	0.98-3.04	1.-50.d	^t^
101.-150.d	192	52	27.1%	1.93	1.11-3.35	1.-50.d	*
>350.d	114	36	31.6%	2.06	1.12-3.79	1.-50.d	*
dry cows	182	50	27.5%	1.67	0.95-2.91	1.-50.d	^t^

number of lactation							
first calvers	293	93	31.7%	1.74	1.22-2.47	>3 completed lactations	**
first calvers	293	93	31.7%	1.51	1.07-2.11	2-3 completed lactations	*

somatic cell count (/ml milk)							
<100,000	1028	279	27.1%	1.57	1.18-2.10	>100,000	**

protein content in milk (%)							
3.00-3.80	906	220	24.3%	1.80	1.06-3.08	<3.00	*
>3.80	198	54	27.3%	2.30	1.18-4.48	<3.00	*

content in urea in milk (in mg/l)							
<150	580	144	24.8%	0.69	0.45-1.06	150-300	^t^

BCS							
3.50-5.00	318	81	25.5%	1.92	1.22-3.04	1.00-3.25	*

presence of a Super-Shedder							
yes	1071	323	30.2%	2.63	1.90-6.64	no	***

*** p-value < 0.001

**: p-value < 0.01

*: p-value < 0.05

^t^: tendency, p-value < 0.1

<: smaller than

>: bigger than

d: day, %: percentage

CI: confidence interval

sign.: significance, p-value

BCS: body condition score according to Edmonson et al. [51]

Additionally, a cow kept in a herd with Super-Shedders had a significant, more than two-fold risk of being positive in the *stx*-detection by PCR compared to a cow in a herd with no Super-Shedder (Table 4).

The *stx *prevalence was highest in the late summer months (August, September, and October) with 27.9%, 28.8% and 35.0% in contrast to the spring months with lower prevalence (February: 20.5%, March: 17.6%, April: 16.3%). In consequence, cows showed a significant higher risk for detection as *stx*-positive in summer, autumn and winter compared to that in spring. Furthermore, cows had a significantly higher risk in autumn for shedding *stx *than in winter (Table 5).

**Table 5:** Seasonality of *stx*-detection in feces of examined cattle expressed in Odds Ratios

	Odds =1			
	
seasons	spring	summer	autumn	winter
spring	X	0.61*	0.43***	0.63*
summer	1.64*	X	0.70^t^	1.02
autumn	2.35***	1.43^t^	X	1.47*
winter	1.60*	0.98	0.68*	X

spring: February, March, April

summer: May, June, July

autumn: August, September, October

winter: November, December, January

*** p-value < 0.001

*: p-value < 0.05

^t^: tendency, p-value < 0.1

## Discussion

Many studies have examined the epidemiology of STEC O157:H7 in cattle populations, but there have been only a few investigations into the relevance and the transferability of these results to other serovars, for instance O26, O91 or O113. These serovars have been detected in cattle herds and are also relevant for human diseases [2,3,20]. Therefore, this study considered STEC in general, not only O157:H7.

Following an evaluation of the shedding patterns of all investigated cows, the strict definition of Super-Shedders with at least four consecutive *stx*-positive samples in the screening PCR and at least half of their samples deemed *stx*-positive was necessary to distinguish intermittent-shedding and persistent-shedding cows. A strictly longitudinal definition of a Super-Shedder as in the present study was also used in the investigations of Lim et al. and Carlson et al. [18,19]. Carlson et al. reasoned this definition by findings of Woerner et al. that the persistence of shedding of O157:H7 is much more important than the STEC count in the feces, as suggested by Chase-Topping et al. in their definition of a Super-Shedder [12,19,21].

Due to the methodological approach which included a step of high dilution (10^-5^-10^-6^) to plate the bacteria for the colony-hybridization, an efficient isolation of STEC gave a hint of high numbers of STEC in the fecal sample. The isolation of STEC in this study mainly was possible in the samples of Super-Shedders, which is congruent with the results mentioned by Chase-Topping et al. who defined Super-Shedders basically about the number of shed STEC [12].

To our knowledge, the study presented here is the first showing Super-Shedding of serovars other than O157:H7 by Super-Shedder dairy cattle.

Compared to the study of Low et al. with 3.9% of all cows identified as Super-Shedders for the serovar O157:H7, the proportion of analyzed Super-Shedders for non-O157:H7 serovars in all investigated herds in this study was high with 10.0% (14 cows) [22]. This is possibly due to the higher colonization of cattle with non-0157:H7-serovars [23,24]. In general, a high proportion of Super-Shedders is associated with a high proportion of low-level and intermittent shedders due to the higher possibility of transmission [22,25]. As previously described for STEC strains in cattle, most of the isolated strains do not possess the *eae *gene [23,26]. However, the mechanisms of adherence for bovine non-O157:H7 STEC-isolates are not restricted to *eae *but also include local or diffuse adherence on HEp2-cells [27]. Further virulence factors of adherence for non-O157:H7 STEC, also associated with human diseases, could comprise Saa (an autoagglutinating adhesion), Iha (adherence-conferring protein similar to *Vibrio cholerae *IrgA), Efa1 (*E. coli* factor for adherence) and LPF (long polar fimbriae; closely related to LPF of *Salmonella enterica *serovar Typhimurium) [28-32]. Concerning Efa1, Stevens et al. proved in *in vivo *experiments the relevance of this virulence factor for the colonization of intestinal mucosa in cattle [29]. As we currently do not know whether Super-Shedding of non-O157:H7 STEC is also caused by enhanced colonization of the terminal rectum, as it has been shown for O157:H7 colonization, future studies clearly should address these questions [33].

Congruent to other literature, a STEC prevalence of 24.7% was detected in healthy dairy cows in Northern Germany in this study [23,26]. In other studies in Germany, values from 18.0% up to 86% were assessed [23,34-36]. In the other parts of Europe, the prevalence was supposed to be slightly lower with percentages of 11.0% to 21.0% [37]. Differences in the obtained prevalence can be related to different methods of detection on the one hand and the sampling method and the sample size on the other hand. The methodology used in the present study and also in the study by Geue et al. with an enrichment step before the PCR and the colony-hybridization resulted in the hitherto highest prevalence of STEC in beef cattle and heifers in Germany [23]. The most sensitive sampling method, at least for STEC O157:H7, seemed to be the rectal swab [38,39]. This is probably due to the colonisation of the recto-anal junction of the intestinal mucosa, which is directly sampled by the swab [16,22].

Only 18 animals (12.9%) of 140 sampled cows were *stx*-negative over the whole period of sampling. Most of the cows were defined as intermittent shedders; they were *stx*-positive in the PCR for short periods of consecutive samplings or for one sampling, but did not reach the number of consecutive *stx*-positive samplings necessary to fulfill the definition of a Super-Shedder.

With regard to the virulence gene patterns, the assessed dominance of *stx*_2 _and *stx*_1 _+ *stx*_2_-positive strains in cattle in the present study is in accordance with other authors [23,26]. This high proportion of *stx*_2_, known as the most virulent Shiga toxin for humans, in bovine STEC strains poses an important risk for humans in contact with cattle and cattle-derived foods. This holds especially true because several cases of STEC infections in humans have been associated with STEC strains without the *eae *gene, but with *stx*_2 _and other factors for adhesion [1,30,40-42].

Based on the technical and methodological efforts of serotyping, only 61 isolates originating in the Super-Shedding cows were examined. These serovars belonged to 24 non-O157:H7 STEC-serovars, partly with previous references of description in healthy cattle but also in healthy and diseased human [43]. This diversity of serovars is typical for cattle; Blanco et al. isolated in 328 cattle 66 STEC that belong to 25 serovars [44]. Three of the most frequent serogroups in the present study were also identified in the investigation of Blanco et al. [44]. Eight of the serovars isolated in the present study have already been associated with diseases in humans [43]. Especially the serovars O8:H19, O8:H21, O22:H8, O113:H21 and Orough:NM were also detected in diseased humans with hemolytic-uremic syndrome (HUS) and thrombotic-thrombocytopenic purpura (TTP), seven other isolated serovars (see Table 3) were isolated in cases of humans with diarrhea [43]. The most predominant serovars O113:NM and O22:H8 induced diarrhea respectively diarrhea and hemolytic-uremic syndrome (HUS) in some cases in humans [43]. The serovar Orough:H2 was not associated with the induction of severe human disease but was characterized by the possession of *stx_2_, EHEC-hly*_*A *_and *eae *genes, which is the typical virulence patterns for the human pathogen EHEC [43].

In the present study, one single cow hosted two to four different serovars. However, this number is limited by the number of colonies taken from the plate. In the study of Blanco et al., up to three different serovars were isolated by some cows [44].

The serotyping of the small proportion of all isolates was sufficient to confirm the hypothesis of Super-Shedders of non-O157:H7 STEC by at least two cows shedding continuously O113:NM and 022:H8. This indicates that also non-O157:H7 serovars are able to colonize the bovine intestinal mucosa, resulting in persistent Super-Shedding. Further studies under defined experimental conditions are needed to investigate the precise colonization and shedding in detail. Regarding the other risk factors, the seasonality for the shedding strains serogroup O157 showed highest prevalence in summer, those for STEC in general a prevalence peak in autumn [45,46]. In accordance with both studies, the highest prevalences in this study were also detected in the late summer months. Thus, saisonality is clearly associated with STEC shedding, regardless of which serotype is tested for.

The higher risk for heifers and for cows with 50 to 150 days in milk can be explained by the metabolic and emotional stress of these animals and actually was shown for serovar O157:H7 [25,47]. The relevance of age for the occurrence of pathogenic *E. coli *has already been presented by Wieler et al. [48]. Regarding O157:H7, only few studies have dealt with the connection of milk content and the shedding of this serovar [11]. These authors clearly showed the impact of milk content, probably related to diet, health and stress, on the shedding of non-O157:H7 STEC as well. Moreover, the presence of a Super-Shedder in the herd as a persistent source of infection was shown to be a risk factor for the shedding of serogroup O157 and was confirmed for non-O157:H7 STEC [12].

## Conclusion

This study confirms the importance of seasonality and Super-Shedders as a major cause of non-O157:H7 STEC shedding in cattle farms. Super-Shedders maintain and aggravate the infectious cycle of STEC in the herd; therefore, interventions at individual animal level might serve as the basis for effective pathogen control. Pathogen virulence profiles, common in human isolates, are frequently found in bovine STEC, corroborating the important reservoir function of cattle as potential human health risks. Risk factors for the excretion of STEC have been examined before; however the situation remains rather complex, and main influencing factors varied due to study design and animal premises analyzed. The importance of Super-Shedders for the intra herd epidemiology of non-O157:H7 STEC should be proven by intervention strategies. Future research approaches should concentrate on the facilitated identification of Super-Shedders and the development of potent vaccines or treatments. These strategies represent the key to an effective interruption of STEC-infection cycles.

## Methods

### Animal selection and sample collection

The study was conducted on six dairy farms in Schleswig-Holstein/Germany in the period of 02/2007 until 01/2008 over twelve months. These farms were chosen out of ten farms that had participated in a previous investigation on the evaluation of the health status of dairy cows in correlation with farm type, diet and herd management [49]. The design of the study was longitudinal with the intent to follow the fecal excretion patterns of STEC of selected cows. Therefore, the distribution of cows in different numbers of lactation was analyzed and, according to this distribution, the cows for sampling were randomly selected on each farm. The necessary number of sampled cows was calculated with the formula suggested by Cannon and Roe, with a limit of significance of 90.0% and an estimated prevalence of 50.0% [50].

The analysis of certain risk factors for the individual animal and for the herd was performed with additional information about herd management, health status and body condition scores [51], which were evaluated on each sampling day. Moreover, data on the amount and content of milk, the number of passed lactations and the days in milk were provided by the regional organization for animal recording ('Landeskontrollverband Schleswig-Holstein'). A number of 1,646 fecal samples were examined, originating from 182 cows. However, 133 of these cows on the six farms were sampled monthly during the whole period of sampling. Animals sampled at least six consecutive times, 140 cows, were considered in the descriptive longitudinal analysis of shedding.

Fecal samples of approximately 20 g were taken by fecal grab with disposal gloves, if possible. Dry cows, kept on pastures in summer, were observed und fresh feces from the pasture were taken immediately after shedding.

### Bacterial culture and identification of STEC

All samples were examined in the laboratory within eight hours of collection. The fecal samples were mixed manually; afterwards a swab was plunged three to five times in the sample. The swab was agitated in a two-milliliter phosphate buffered saline-solution for one minute. Fifty micro liters of this dilution were added to three milliliters Luria-Bertani (LB)-bouillon and incubated over night at 150 rpm by 37°C. Following to this enrichment step, DNA of each sample was prepared by boiling for 20 min, and screened with the primers MK1 and MK2 according to the PCR published by Karch and Meyer [52]. Fifty micro liters of the Shiga toxin gene-positive samples and 100 μl of a 1 × 10^-5^-fold dilution and 100 μl of a 1 × 10^-6^-fold dilution were plated on LB-agar-plates. After 18 h of incubation, plates with equally distributed single colonies in a number of 100 to 500 were examined by colony-hybridization using the DIG-Kit (Roche Diagnostics GmbH, Mannheim/Germany) following the manufacturers protocol to isolate STEC. The probe was synthesized by PCR-labeling with the PCR-DIG labeling set (Roche Diagnostics) and the primers MK1 and MK2 mentioned above [52]. Up to 20 identified STEC-colonies per sample were isolated and characterized by PCR for the virulence factors *stx*_1_, *stx*_2_, *eae *(intimin) and *EHEC-hly*_*A*_(EHEC-hemolysin_A_) with primers described before [53-56]. Furthermore, representing the Super-Shedders on farm level three Super-Shedding cows of three farms with Super-Shedders were selected. On another farm, three Super-Shedders were chosen to indicate present serotypes on herd level. From of all 278 STEC isolated from these six Super-Shedders, groups were formed, classified by virulence patterns, sampling date and sampled animal, followed by a random selection of one STEC-isolate per group. This resulted in 61 STEC-isolates which were tested serologically by the Robert Koch Institute following the protocol of Ørskov and Ørskov [57]. In this study, cows were defined as Super-Shedders if at least half of their samples and equal or more than four consecutive samplings were classed as *stx-*positive.

### Data analysis

The data were statistically analyzed using the program 'Excel' (Windows XP, Microsoft Office Excel 2003) and the procedure 'logistic' in 'SAS' (SAS^® ^9.1, SAS Institute Inc, Cary, NC, USA) calculating Odds Ratios (OR). Comparisons with p-values < 0.05 were considered as statistically significant; p-values between 0.05 and 0.10 were indicated as statistical tendency.

The selected model included the following variables: the results of the screening PCR according to Karch and Meyer in the specification of zero - corresponding to no *stx *detected in the feces of the sampled cow - and one - corresponding to *stx *detected in the feces of the sampled cow - were used as dependent variables [52]. As independent variables, the month of sampling (1 for January, 2 for February, 3 for March and so on), and the farm and the number of lactation were considered. All sampled cows were categorized in three classes by their number of lactation: the first class included the first calving cows, the second class the cows in the second and third lactation, and the third class all cows that completed more than three lactations. Another independent variable was the number of days in milk (DIM), classified in classes of 50 days (DIM-Class 1 = cows in 1^st ^to 50^th ^day of lactation, DIM-Class 2 = cows in 51^st ^to 100^th ^day of lactation and so on; DIM-class 8 = all cows in lactation for more than 350 days, DIM-class 9 = all dry cows). Furthermore, the model included the presence of Super-Shedding cows in the herd. Cows with at least half of their samples and at least four consecutive *stx*-positive samples in the screening PCR were defined as Super-Shedding cows using the strictly longitudinal definition. Because of the limited sample size, each additional variable such as milk yield, milk contents and body condition score (BCS) was added separately to the model in single steps and the respective odds ratios were estimated.

## Competing interests

The authors declare that they have no competing interests.

## Authors' contributions

AM carried out all samplings and the bacteriological analyses and draft the manuscript. LW made substantial contributions to the interpretation of the results. KH and TS provided support in the validation of results. LW, KH and TS critically revised the manuscript. AF managed the serotyping of the STEC-isolates. NK designed and coordinated the study and was involved in drafting the manuscript.

All authors read and approved the final manuscript.
